# Extended stereotactic brain biopsy in suspected primary central nervous system angiitis: good diagnostic accuracy and high safety

**DOI:** 10.1007/s00415-020-10157-2

**Published:** 2020-08-19

**Authors:** Veit Michael Stoecklein, Lars Kellert, Maximilian Patzig, Clemens Küpper, Armin Giese, Viktoria Ruf, Jonathan Weller, Friedrich-Wilhelm Kreth, Florian Schöberl

**Affiliations:** 1 Department of Neurosurgery, Ludwig-Maximilians-University Munich, Munich, Germany; 2 Department of Neurology, Ludwig-Maximilians-University Munich, Marchioninistr.15, 81377 Munich, Germany; 3 Department of Neuroradiology, Ludwig-Maximilians-University Munich, Munich, Germany; 4 Center for Neuropathology and Prion Research, Ludwig-Maximilians-University Munich, Munich, Germany

**Keywords:** Primary angiitis of the central nervous system, Stroke, Cerebral vasculitis, Brain biopsy, Stereotactic neurosurgery, Image-guided neurosurgery

## Abstract

**Objective:**

To evaluate the diagnostic accuracy and safety of extended stereotactic brain biopsy (ESBB) in a single center cohort with suspected primary angiitis of the central nervous system (PACNS).

**Methods:**

A standardized stereotactic biopsy targeting MRI-positive lesions and collecting samples from the meninges and the cortex as well as from the white matter was performed in 23 patients with clinically suspected PACNS between 2010 and 2017. The relationship between biopsy yield and clinical characteristics, cerebrospinal fluid parameters, MR-imaging, time point of biopsy and exact localization of biopsy as well as number of tissue samples were examined.

**Results:**

PACNS was confirmed in 7 of 23 patients (30.4%). Alternative diagnoses were identified in 7 patients (30%). A shorter time period between the onset or worsening of symptoms (*p* = 0.018) and ESBB significantly increased the diagnostic yield. We observed only minor and transient postoperative complications in 3 patients (13.0%). ESBB led to a direct change of the therapeutic regime in 13 of 23 patients (56.5%). Careful neuropathological analysis furthermore revealed that cortical samples were crucial in obtaining a diagnosis.

**Conclusion:**

ESBB is a safe approach with good feasibility, even in critically ill patients, and high diagnostic accuracy in patients with suspected PACNS changing future therapies in 13 of 23 patients (56.5%). Early biopsy after symptom onset/worsening is crucial and (sub)acute MRI-lesions should be targeted with a particular need for biopsy samples from the cortical layer.

## Introduction

Primary angiitis of the central nervous system (PACNS) is a rare inflammatory disorder with an estimated annual incidence rate of 2–3 per one million individuals [1, 2]. Inflammation and consecutive destruction of medium- or small-sized brain vessels in the brain and spinal cord lead to ischemia and diverse progressive neurological deficits [3]. The diagnostic workup of PACNS is challenging, because the clinical symptoms are heterogenous and nonspecific [4, 5]. In the absence of a tissue sample, PACNS is mainly a diagnosis of exclusion. Diagnosis of PACNS can be assumed if new neurological symptoms are present that cannot be explained otherwise, secondary causes of angiitis are excluded and cerebral angiogram and/or biopsy show pathological findings consistent with PACNS [4]. The likelihood of correct diagnosis is increased if there are additional findings consistent with angiitis on ancillary testing, e.g. multiple ischemic or contrast enhancing lesions on MRI and inflammatory CSF changes. Notably, similarly pathological CSF exams and MRI findings also occur in other neurologic diseases like CNS-lymphoma or infectious CNS disease [6].

Brain biopsy, albeit discussed controversially, remains the gold standard for diagnosis of PACNS. Tissue sampling from the meninges, the cortical surface and the deep white matter is considered the most accurate way to achieve a definite diagnosis [3, 4, 7]. Criticism of this approach stems from alleged high false negative rates (I) invasiveness of the procedure, (II) the notion that biopsies cannot be easily repeated multiple times in one individual, (III) and lack of standardized protocols (IV) [2].

Since therapy of PACNS requires long lasting and aggressive immunosuppressive therapy, reliability, validity and safety of the diagnostic procedure are of utmost importance for the individual patient. A definition of how and where to perform a brain biopsy in patients with suspected PACNS has not been presented yet. Open, frame-less craniotomy techniques and stereotactic, frame-based approaches have been proposed. It furthermore remains unclear, whether biopsy of the cortical surface or of deep-seated white matter lesions is more likely to yield a positive histological result for PACNS or differential diagnoses.

Here, we tested a standardized stereotactic, frame-based biopsy technique. Samples from the three relevant anatomical compartments, i.e. meninges, cortex and white matter, were obtained by sequential tissue sampling along the trajectory that targeted the MRI-positive lesion (extended stereotactic brain biopsy; ESBB). Stereotactic tissue sampling was preceded by non-stereotactic superficial tissue sampling of the meninges and cortex directly after burr hole trepanation. The procedure was designed to be as minimally invasive as possible, making even seriously ill patients eligible for the procedure. We set out to assess safety and diagnostic accuracy of this approach in a single-center cohort of patients with unclear neurological symptoms and non-diagnostic MRI lesions. We furthermore examined patient and treatment related variables in order to identify predictive factors for positive biopsy results.

## Methods

### Chart analysis

We searched the electronic medical record at the Hospital of the Ludwig-Maximilians University, Munich, Germany, for patients with suspected PACNS. 23 Patients who underwent extended stereotactic brain biopsy (ESBB) with cortical biopsy for clinically suspected or at least possible PACNS between 2010 and 2017 were identified and retrospectively analyzed. Clinical work-up of all the patients included MR-imaging [diffusion-weighted imaging (DWI), fluid-attenuated inversion recovery (FLAIR), T2, susceptibility-weighted imaging (SWI), T1 pre- and post-contrast)], MRI-based angiography (time-of-flight and contrast enhanced MR-angiography), CSF-analysis (cell count and cell differentiation, protein and glucose levels and oligoclonal bands) and laboratory tests including systemic vasculitis parameters such as ANA-levels and -differentiation as well as ANCA-levels and rheumatoid factor. Furthermore, serological examinations and CSF-analyses for borrelia species, HIV, hepatitis A, B, C, VZV, HSV and TBC as well as evaluation for vasculitic skin and/or ophthalmological changes were performed in each patient. All patients with old and acute ischemic brain lesions underwent cardiac examination including long-term electrocardiogram for 72 h and cardiac ultrasound examination. Conventional catheter angiography was performed in 12/23 patients.

Invasive brain biopsy was considered indicated when non-invasive testing provided inconclusive results but PACNS was still suspected or considered likely according to pertinent diagnostic criteria. We obtained data regarding (1) demographics; (2) CSF findings; (3) MRI findings and MR angiography and/or catheter angiography findings; (4) time point of brain biopsy in relation to symptom onset and/or worsening; (5) spatial relation of specimens obtained along the stereotactic track to MRI-positive lesions (DWI-lesions and/or contrast enhancing lesions) and specimen size; and (6) postoperative complications. Furthermore, therapy and 1-year clinical outcome was retrospectively evaluated by means of the National Institutes of Health Stroke Scale (NIHSS) and the modified Rankin Scale (mRS). The therapeutic regimes of each patient after brain biopsy were taken from the medical reports available (*n* = 23). The NIHSS and mRS were calculated in each patient with histologically proven PACNS (*n* = 7) or clinically still presumable PACNS despite unspecific, non-diagnostic histological findings (*n* = 5) at initial presentation and after one year follow-up by analyzing standardized neurological examination from the medical reports of each patient.

### Operative procedure

A frame-based multimodal imaging-guided stereotactic biopsy technique was used in all patients. Imaging included localized contrast-enhanced CT and additionally contrast-enhanced T1 and T2-weighted MRI sequences as well as a contrast-enhanced MR angiography, which were automatically fused with the localized CT data. For trajectory planning, a workstation was used allowing triplanar simulation of any trajectory. An MRI-positive lesion was always targeted. A lesion was considered acute if it showed restricted diffusion and/or contrast enhancement. Small tissue samples (1 mm^3^) were collected in one-millimeter steps along the biopsy track from the brain surface towards the lesion. Additionally, multiple samples were collected with a biopsy forceps directly from the cortical surface in the direct vicinity of the trajectory. ESBB was conducted using a 1 cm burr hole.

### Neuropathological analysis

Stereotactic biopsy samples of a maximum size of 1 mm^3^ were fixed with 4% neutral buffered formalin (Microcos GmbH, Garching, Germany), paraffin-embedded and routinely stained for Hematoxylin and Eosin (H&E), Elastica van Gieson (EvG), Periodic acid-Schiff stain (PAS) and Gomori (Gom). Subtypes of leucocytes were differentiated by immunohistochemical staining against surface antigens CD3 (polyclonal, 1:50, Agilent Technologies, Santa Clara, CA, USA), CD20 (L26, 1:100, Agilent Technologies), CD45 (2B11 + PD7/26, 1:400, Bio SB, Santa Barbara, CA, USA) and CD68 (KP1, 1:100, Agilent Technologies). In case of suspected cerebral amyloid angiopathy (CAA), Congo red stain and immunohistochemical staining against beta-Amyloid (4G8, 1:5000, Covance, Princeton, NJ, USA) were performed in addition.

Stained slides of tissue samples from 23 cases were systematically reviewed for the morphological criteria of vasculitis: Trans/intramural inflammatory infiltrate and destruction of the vascular wall of intraparenchymal vessels. As PACNS typically affects small vessels with a diameter of less than 100 µm, the full picture of vascular pathology is usually well depicted within a biopsy sample, if present. Thus, the small biopsy specimens of stereotactic biopsies ought to be sufficient to make a histological diagnosis of CNS vasculitis, accurate sampling provided.

### Statistics

For quantitative comparisons between groups the Chi square test was used for nominal variables and the Mann–Whitney *U* test was applied for ordinal variables. *p* values below 0.05 were considered significant.

### Ethics

The study was approved by the local ethics commission of the Ludwig-Maximilians University (Nr. 17-174) and was performed in accordance with the ethical standards laid down in the 1964 Declaration of Helsiniki and ist later ammendments.

## Results

Between 2010 and 2017, 23 patients (11 females, 48%) received ESBB for clinically suspected or at least possible PACNS according to current guidelines and after thorough clinical work-up without a definitive diagnosis. A stereotactic brain biopsy of an ischemic or contrast enhancing lesion was conducted in each patient (*n* = 23; 100%), an additional cortical surface and meningeal biopsy in 19 patients (83%). In 10 patients (43%) an acute lesion (either DWI-positive or contrast enhancing) was the exact biopsy localization, in 13 patients (57%) older, already DWI-negative, FLAIR-positive ischemic lesions.

In 7/23 patients (30%) a definite diagnosis of PACNS was made histologically. Thereof, ß-amyloid related angiitis (ABRA) was found in 2, lymphocytic vasculitis in 4 and granulomatous vasculitis in 1. A different and definite diagnosis could be made in 7/23 patients (30%). These diagnoses included cerebral amyloid angiopathy with/without inflammation (CAA; *n* = 3), encephalitis, intravascular diffuse large B cell lymphoma of the CNS, a demyelinating lesion and encephalitis (*n* = 1 each). No definite histopathological diagnosis could be made in 9/23 cases (39%). In one of these cases, even though a definite histopathological diagnosis of PACNS could not be made, the histopathological results were suspect of PACNS. Although the biopsy was non-diagnostic with unspecific results (i.e. gliosis with unspecific inflammatory changes), in 5 of these 9 cases (21.7%), PACNS was still very likely and immunosuppressive treatment was initiated. In the remaining 4 cases the clinical course and additional tests (i.e. extended and repeated laboratory tests, repeated brain-MRI, skin biopsy) made other diagnoses than PACNS more likely, which was posterior reversible encephalopathy syndrome (PRES), Ehrmann Sneddon´s syndrome, primary antiphospholipid syndrome (APS) and unclassified systemic vasculitis with CNS-involvement. These cases represent true-negative cases.

Clinical (i.e. headache, focal neurological deficits etc.) characteristics, CSF findings, MRI findings and conventional angiography could not reliably differentiate between patients with biopsy proven PACNS and patients with a definite alternative diagnosis or unspecific histological changes in brain biopsy (Table 1). Headache seemed to be more present in patients with confirmed PACNS (57%) as compared to the control group without vasculitis (19%) (*p* = 0.066). Only one patient received immunosuppressive therapy with high-dose methylprednisolone before ESBB.

**Table 1:** Whole study group (*n* = 23): Demographics, clinical characteristics, CSF, MRI and conventional angiography results & biopsy variables

	All patients (*n* = 23)	Biopsy positive for PACNS (*n* = 7)	Biopsy negative for PACNS (*n* = 16)	*P* value
Age (Mean ± SD)	57.5 ± 16.5	67 ± 10	53.0 ± 17.0	0.051
Sex				0.752
Male (%)	12 (52.2%)	4 (57.1%)	8 (50%)	
Female (%)	11 (47.8%)	3 (42.9%)	8 (50%)	
Clinical characteristics				
Headache (%)	7 (30.4%)	4 (57.1%)	3 (18.8%)	0.066
Focal deficits (%)	20 (87.0%)	7 (100%)	13 (81.3%)	0.22
Cognitive/behavioral changes (%)	12 (52.2%)	3 (42.9%)	9 (56.3%)	0.55
Epilepsy	3 (13.0%)	2 (28.6%)	2 (12.5%)	0.54
Decreased vigilance state (GCS < 15) (%)	2 (8.7%) (GCS 13 each)	0 (0%)	2 (12.5%)	0.33
CSF results				
WBC (cells/mm^3^) (Median)	2 (0–56)	2 (1–56)	2 (0–25)	0.14
Protein (mg/dl) (Median)	50 (30–266)	62 (39–266)	45 (31–250)	0.11
Glucose (mg/dl) (Median)	65 (54–84)	65 (54–81)	63 (58–84)	0.75
OCBs %	6 (26%)	3 (43%)	3 (19%)	0.31
MRI findings				
Acute ischemic infarction (%)	16 (69.6%)	5 (71.4%)	10 (62.5%)	0.90
Old ischemic infarction (%)	15 (65.2%)	5 (71.4%)	10 (62.5%)	0.68
WML (%)	15 (65.2%)	5 (71.4%)	10 (62.5%)	0.33
Macrobleeds (%)	2 (8.7%)	0 (0%)	2 (12.5%)	0.14
Microbleeds (%)	8 (34.8%)	4 (57.1%)	4 (25%)	0.35
Sulcal siderosis/SAH (%)	4 (17.4%)	2 (28.6%)	2 (12.5%)	0.39
Contrast enhancing lesions (%)	9 (39.1%)	5 (71.4%)	4 (25%)	0.10
Brain biopsy variables				
Number of specimens (cortical surface) (Median)	10 (0–24)	8 (0–23)	10 (0–24)	0.27
Number of specimens (stereotactic trajectory) (Median)	11 (3–21)	13 (7–21)	10 (3–21)	0.19
Time point of biopsy (days after symptom onset/worsening; median)	5 (2–90)	4 (2–5)	22 (2–90)	0.018*
Exact biopsy localisation (acute lesion) (%)	10 (43.5%)	6 (85.7%)	4 (25%)	0.28

*MRI* magnetic resonance imaging, *CSF* cerebrospinal fluid, *WBC* white blood cells, *OCBs* oligoclonal bands, *WML* white matter lesions

In 6 cases a definite diagnosis could be made both from samples taken along the stereotactic track and from samples taken by cortical/meningeal biopsy (44% of positive cases). In 6 cases, only samples obtained stereotactically revealed the diagnosis (43%), but only in 2 cases (14%) the diagnosis was made based on samples obtained by direct cortical/meningeal biopsy only. Thus, despite lacking statistical significance, there was a trend towards better diagnostic accuracy in samples obtained stereotactically (*p* = 0.084). Considering only PACNS cases, we found that PACNS was diagnosed based on samples taken along the stereotactic track in 5 cases (71% of PACNS cases). Samples taken both along the stereotactic track and by direct cortical/meningeal biopsy formed the basis for the diagnosis in 1 PACNS case. PACNS was diagnosed based only on cortical/meningeal samples in 1 case. However, careful neuropathological review of the samples revealed that samples from the cortical layer, rather than from the white matter, either obtained stereotactically or by direct cortical biopsy, were crucial in all cases for making the diagnosis.

Differential diagnoses were made based on samples obtained stereotactically in 1 case, on samples obtained both stereotactically and by direct cortical biopsy in 5 cases and by cortical biopsy only in 1 case.

Biopsy of acute lesions (either DWI-positive or contrast enhancing) did not show a higher accuracy as compared to older, post-ischemic lesions (*p* = 0.28). However, the time point of biopsy was critical, since diagnostic accuracy significantly improved, when the tissue samples were taken within the first 5 days after symptom onset or worsening (*p* = 0.018) (Table 2). The sample size was comparable in all cases (1 mm^3^) due to a standardized operative approach in all cases with use of a 1 mm biopsy forceps. The mean number of tissue samples obtained from the stereotactic trajectory was 15 (range 3–21) and the mean number of samples taken from the cortical surface was 10 (range 0–24). The number of samples did not differ significantly between patients with and without histologically confirmed PACNS.

**Table 2:** Subgroup analysis (histologically confirmed vs. suspected biopsy-negative PACNS): demographics, clinical characteristics, CSF, MRI and conventional angiography results & biopsy variables

	All PACNS patients (*n* = 12)	Histologically confirmed PACNS (*n* = 7)	Suspected, biopsy-negative PACNS (*n* = 5)	*P* value
Age (Mean ± SD)	59.1 ± 12.9	67 ± 10.4	48 ± 5.6	0.015*
Sex				0.80
Male (%)	6 (50%)	3 (42.9%)	2 (40%)	
Female (%)	6 (50%)	4 (57.1%)	3 (60%)	
Clinical characteristics				
Headache (%)	4 (33.3%)	4 (57.1%)	0 (0%)	0.038*
Focal deficits (%)	12 (100%)	7 (100%)	5 (100%)	1
Cognitive/behavioral changes (%)	4 (33.3%)	3 (42.9%)	1 (20%)	0.41
Epilepsy	2 (16.7%)	2 (16.7%)	0 (0%)	0.19
Decreased vigilance state (GCS < 15) (%)	0 (0%)	0 (0%)	0 (0%)	1
CSF results				
WBC (cells/mm^3^) (Median)	1.5 (0–56)	2 (1–56)	1 (0–4)	0.51
Protein (mg/dl) (Median)	49.5 (31–266)	62 (39–266)	35 (30–56)	0.015*
Glucose (mg/dl) (Median)	65.5 (54–81)	65 (54–81)	66 (59–70)	0.57
OCBs %	6 (50%)	3 (42.9%)	3 (60%)	0.12
MRI findings				
Acute ischemic infarction (%)	10 (83.3%)	5 (71.4%)	5 (100%)	0.19
Old ischemic infarction (%)	10 (83.3%)	5 (71.4%)	5 (100%)	0.19
WML (%)	7 (58.3%)	5 (71.4%)	2 (40%)	0.52
Macrobleeds (%)	0 (0%)	0 (0%)	0 (0%)	0
Microbleeds (%)	3 (25%)	4 (57.1%)	0 (0%)	0.038*
Sulcal siderosis/SAH (%)	2 (16.7%)	2 (28.6%)	0 (0%)	0.19
Contrast enhancing lesions (%)	3 (35%)	3 (42.9%)	0 (0%)	0.09
Brain biopsy variables				
Number of specimens (cortical surface) (Median)	9 (0–23)	8 (0–23)	14 (8–16)	0.46
Number of specimens (stereotactic trajectory) (Median)	15.5 (7–21)	13 (7–21)	21 (9–21)	0.14
Time point of biopsy (days after symptom onset/worsening; median)	5 (2–45)	4 (2–5)	30 (22–45)	0.004**
Exact biopsy localisation (acute lesion) (%)	6 (50%)	6 (83.3%)	0 (100%)	0.0034**

*MRI* magnetic resonance imaging, *CSF* cerebrospinal fluid, *WBC* white blood cells, *OCBs* oligoclonal bands, *GCS* Glasgow Coma Scale, *SAH* subarachnoid hemorrhage, *WML* white matter lesions

Direct comparison of the patients with biopsy-proven PACNS (*n* = 7) with the subgroup with still suspected PACNS despite non-diagnostic brain biopsy showing only unspecific gliosis and inflammation (*n* = 5) revealed the following differences: The mean age in the biopsy-proven PACNS patients was significantly higher (*p* = 0.015), the CSF-protein levels were higher in this subgroup (*p* = 0.015) and the rate of patients with headache (*p* = 0.038) and microbleeds (*p* = 0.038) was also higher in this subgroup (see Table 2). The biopsy time point was significantly delayed in the subgroup with suspected PACNS, but only unspecific and thus non-diagnostic brain biopsy results (*p* = 0.004). Correspondingly, less often acute lesions (i.e. either DWI-positive or contrast-enhancing lesions) were the target of brain biopsy in this subgroup (*p* = 0.034) (see Table 2).

### Adverse events

Wound infection requiring operative revision occurred in 1 patient. Another patient suffered from postoperative delirium. In one case, asymptomatic minor bleeding occurred at the biopsy site which did not require intervention. No further postoperative complications occurred. In total, transient morbidity occurred in 13.0% of cases. No permanent morbidity or procedure-related mortality was observed.

### Therapy and clinical course

Altogether, the results obtained from the brain biopsy directly changed the therapeutic regime in 13 of the 23 patients (56.5%), which were the 7 patients with confirmed PACNS and 6 of the 7 patients with a definite diagnosis other than PACNS (CAA-ri *n* = 2; intravascular lymphoma *n* = 1; GvHD-encephalitis *n* = 1; tumefactive multiple sclerosis *n* = 1; neurotuberculosis with secondary vasculitis *n* = 1). In addition, in another 2 cases with only unspecific histopathological findings (i.e. gliosis and unspecific inflammatory changes) the brain biopsy results guided further diagnostics thus leading to other diagnoses than PACNS such as Ehrmann–Sneddon´s syndrome and primary antiphospholipid antibody syndrome. In 5/9 patients PACNS remained the most likely differential diagnosis despite only unspecific gliosis and inflammatory findings obtained by brain biopsy. Due to recurrent ischemic events in these patients with concomitant morbidity immunosuppression with either high-dose steroids alone (i.e. methylprednisolone 5 × 1000 mg) followed by oral tapering of steroids (*n* = 3), high-dose steroids plus cyclophosphamide (*n* = 1) or high-dose steroids plus azathioprine (*n* = 1) was started (see Table 3). The 7 patients with definite PACNS, as confirmed by ESBB, were treated either with high-dose steroids plus cyclophosphamide (*n* = 5) or high-dose steroids in combination with azathioprine (*n* = 2) (see Table 3).

**Table 3:** Overview of the 23 cases with biopsy results, final clinical diagnoses and immunosuppression/specific therapies

Patient (Nr.)	Diagnosis brain biopsy	Final diagnosis	Immunosuppression/specific therapy
1	ß-amyloid-related small vessel vasculitis	ABRA	Cyc. plus high-dose steroids
2	lymphocytic small vessel vasculitis	PACNS	Cyc. plus high-dose steroids
3	granulomatous small vessel vasculitis	PACNS	Cyc. plus high-dose steroids
4	ß-amyloid-related small vessel vasculitis	ABRA	Cyc. plus high-dose steroids
5	lymphocytic small vessel vasculitis	PACNS	Cyc. plus high-dose steroids
6	lymphocytic small vessel vasculitis	PACNS	Aza. plus high-dose steroids
7	Lymphocytic small vessel vasculitis	PACNS	Aza. plus steroids
8	Cerebral amyloid angiopathy with perivascular inflammation	CAA-ri	High-dose steroids
9	ß-amyloid angiopathy	CAA	None
10	Intravascular diffuse large B-cell lymphoma	Intrasvascular lymphoma	Chemotherapy (R-CHOP)
11	Encephalitis with T-cell and macrophage infiltrates	GvHD-encephalitis	Intravenous immunglobulines
12	Cerebral amyloid angiopathy with perivascular inflammation	CAA-ri	High-dose steroids
13	Tuberculous granulomas and vasculitis	Neurotuberculosis and vasculitis	Antibiotics plus oral steroids
14	Demyelinating CNS-inflammation	MS, tumefactive	High-dose steroids
15	Unspecific gliosis	PRES	None
16	Unspecific gliosis	Systemic vasculitis with CNS-involvement	Cyc. plus high-dose steroids
17	Unspecific gliosis	Ehrmann-Sneddon´s syndrome	None
18	Unspecific gliosis	APS	None
19	Unspecific gliosis	PACNS, presumable	High-dose steroids
20	Unspecific gliosis	PACNS, presumable	High-dose steroids
21	Unspecific gliosis	PACNS, presumable	High-dose steroids
22	Unspecific gliosis	PACNS, presumable	Aza. plus high-dose steroids
23	Unspecific gliosis	PACNS, presumable	Cyc. plus high-dose steroids

*ABRA* amyloid ß-related angiitis, *APS* antiphospholipid antibody syndrome, *Aza* azathioprine, *CAA* cerebral amyloid angiopathy, *CAA-ri* cerebral amyloid angiopathy-related inflammation, *Cyc* cyclophosphamide, *GvHD* graft versus host disease, *MS* multiple sclerosis, *PACNS* primary angiitis of the central nervous system, *PRES* posterior reversible encephalopathy syndrome

The overall outcome was favorable in the 7 patients with histologically confirmed PACNS as well as the remaining 5 cases with presumable PACNS but without diagnostic biopsy results (see Table 4). One patient with confirmed PACNS died within one year due to suicide as a direct consequence of organic affective disorder despite psychiatric treatment. All other patients stabilized under immunosuppression and improved clinically as measured by NIHSS as well as in terms of their functional independence in everyday life as measured by mRS (see Table 4). No severe adverse events of immunosuppression were observed.

**Table 4:** Outcome measures in all the 12 patients with immunosuppressive treatment due to either histologically confirmed or clinically suspected PACNS

Patient (Nr.)	Final diagnosis	Immunosuppression	Outcome NIHSS initial/12 months mRS initial/12 months
1	ABRA (biopsy-based)	Cyc. plus high-dose steroids	NIHSS 4/0 mRS 2/0
2	PACNS (biopsy-based)	Cyc. plus high-dose steroids	NIHSS 8/death mRS 3/death
3	PACNS (biopsy-based)	Cyc. plus high-dose steroids	NIHSS 7/2 mRS 3/1
4	ABRA (biopsy-based)	Cyc. plus high-dose steroids	NIHSS 7/3 mRS 3/2
5	PACNS (biopsy-based)	Cyc. plus high-dose steroids	NIHSS 5/1 mRS 3/1
6	PACNS (biopsy-based)	Aza. plus high-dose steroids	NIHSS 7/3 mRS 3/1
7	PACNS (biopsy-based)	Aza. plus steroids	NIHSS 6/3 mRS 3/1
8	PACNS, presumable	High-dose steroids	NIHSS 7/2 mRS 3/1
9	PACNS, presumable	High-dose steroids	NIHSS 10/6 mRS 3/2
10	PACNS, presumable	High-dose steroids	NIHSS 9/6 mRS 3/2
11	PACNS, presumable	Aza. plus high-dose steroids	NIHSS 6/3 mRS 2/1
12	PACNS, presumable	Cyc. plus high-dose steroids	NIHSS 7/3 mRS 3/1

*NIHSS* National Institute of Health Stroke Scale, *mRS* modified Rankin Scale

## Discussion

PACNS comprises a heterogenous group of rare, inflammatory vessel disorders affecting the brain and spinal cord. Brain biopsy is considered the diagnostic gold standard, but there is no consensus on timepoint, surgical method and anatomical location of tissue sampling. Some authors question the indication of brain tissue sampling due to alleged low diagnostic yield and surgery-associated complication rates. Addressing these uncertainties, we investigated systematic stereotactic brain biopsies of different compartments in angiography-negative patients with MRI lesions and clinical findings consistent with PACNS. Lesions were targeted utilizing a frame-based stereotactic approach and biopsies were taken consecutively beginning with the meninges, continuing with the cortex and finally acquiring specimens along the trajectory targeting the deeper-seated, MRI-positive lesions. Patient-related data and clinical parameters were analyzed and correlated with the diagnostic yield of the procedure.

Out of 23 patients (100%) meeting the criteria mentioned above, brain tissue sampling led to definite diagnosis of PACNS or differential diagnoses in 14 (61%) patients. Definite diagnosis of PACNS was determined histologically in 7 (30%). Although biopsy showed only unspecific inflammatory changes and gliosis without a definite diagnosis of PACNS in another 5 (22%) individuals, PACNS was suspected due to the clinical course with recurrent ischemic strokes and after thorough and repeated exclusion of alternative diagnoses. Investigation of these two groups showed that the timeframe between symptom onset or worsening and stereotactic biopsy in patients with histological diagnosis of PACNS was significantly shorter than in the subgroup that was diagnosed based on clinical symptoms and imaging features (*p* = 0.018). This also held true for patients receiving alternative histological diagnoses, suggesting that early tissue sampling after clinical presentation is pivotal. Furthermore, acute, i.e. diffusion-positive or contrast-enhancing, lesions were targeted in 83% of later histologically confirmed PACNS cases. On the contrary, all 5 clinically diagnosed patients that had negative biopsy results displayed MRI-patterns of chronic or subacute lesions, underwent stereotactic biopsy significantly later after clinical deterioration, presented with lower total CSF-protein and less headache. Altogether, this suggests a dormant phase of the disease at the time of stereotactic brain biopsy which might have led to negative biopsy results.

Our biopsy technique involved stereotactic tissue sampling but also non-stereotactic superficial tissue sampling from the meninges and cortex that was conducted directly after burr hole trepanation. Comparison of the respective diagnostic accuracy showed that diagnosis could be made based on either approach in 44% of positive cases. In 43% of patients, only specimens obtained stereotactically led to a diagnosis. Open and non-stereotactic cortical layer biopsy alone was crucial for diagnosis in 14%. Thus, despite lacking statistical significance, there was a trend towards better diagnostic accuracy in samples obtained stereotactically (*p* = 0.084). In 7 (30%) out of 23 patients, PACNS was confirmed histologically. Differential diagnoses could be confirmed in additional 7 patients and comprised cerebral amyloid angiopathy with/without inflammation (CAA; *n* = 3), infectious encephalitis with vasculitis, intravascular diffuse large B cell lymphoma of the CNS, a demyelinating lesion and GvHD-encephalitis (*n* = 1 each).

We analyzed histologically confirmed PACNS cases and compared diagnostic yield of white matter specimens versus cortical specimens, irrespective of whether obtained stereotactically of non-stereotactically. Thorough neuropathological investigation of the tissue with confirmed PACNS showed, that the typical histopathological pattern of PACNS was more prevalent in the cortical layer and essential for diagnosis in all PACNS cases. In most cases, stereotactic brain biopsy provided pivotal cortical tissue (6 out of 7 patients, 86%). In one case, direct non-stereotactic tissue sampling of the cortex alone let to definite diagnosis. Despite this lower yield, we still propose including direct, non-stereotactic biopsies in addition to the stereotactic approach, since stereotactic tissue sampling does not necessarily guarantee obtaining cortical layer specimens.

We report a complication rate of 13% in our cohort with only transient morbidity and no major complication. Stereotactic biopsy led to a therapy change in 57% of cases and prevented initiation of immunosuppressive treatment in 9%. In conclusion, there were only minor complications associated with this minimally invasive surgical procedure and histological analyses had great impact on further treatment regimens.

Successfully diagnosing PACNS surgically remains challenging. Results vary widely, with diagnostic yields being in line with our findings or considerably lower. In a recently published case series of patients with unclear neurological decline who underwent brain biopsies, PACNS was only confirmed histologically in 2 out of 16 patients with clinically suspected PACNS [8]. A recent case series including 79 patients with suspected PACNS who underwent brain biopsy showed histopathological confirmation of PACNS in only 11% of cases with alternative diagnoses in 30% [9]. This very low diagnostic yield for brain biopsies in suspected PACNS was contradicted by a meta-analysis of studies which dealt with brain biopsies for unclear neurological syndromes [10]. Here, the authors found an overall diagnostic success rate of 75% in patients with suspected PACNS with a histological diagnosis of PACNS in 34% of patients. This was based on 3 studies with a total of 106 patients [11–13]. This is in line with our finding of a total diagnostic yield of 60% and a histopathological confirmation rate for PACNS of 30%. The wide range of diagnostic success rates in these studies might be attributed to inconsistent diagnostic and heterogeneous operative approaches. Additionally, PACNS comprises different subgroups of inflammatory vessel diseases and can be stratified according to angiography-negativity with respective biopsy-positivity and vice versa [14]. The former group represents a PACNS type most likely predominantly affecting small vessels that usually cannot be visualized on conventional angiogram [14]. Due to this multifactorial heterogeneity, it is in our view important that biopsies in patients with possible or suspected PACNS are conducted in a standardized and consistent manner across all cases.

In addition to false-negative cases of PACNS due to missing or insufficient brain biopsy, we also have to discuss the important issue of false-positive PACNS-diagnoses. To successfully avoid false-positive diagnoses of PACNS, a clearly defined, standardized and stepwise diagnostic algorithm in all such cases with possible PACNS is mandatory [15–17]. The list of diseases or conditions mimicking PACNS, either when medium-sized vessels or small-vessels are affected, is extensive and most of such alternative diagnoses can be successfully made without brain biopsy/histology [14, 17]. This is of particular clinical relevance, since dispensable immunosuppressive treatments with its multiple side effects and potentially serious complications due to a false-positive diagnosis of PACNS should be avoided [16]. Even technological advances in imaging techniques in the last decade such as black blood based vessel wall MRI of intracranial arteries allows no clear differentiation between distinct conditions such as advanced atherosclerosis, reversible cerebral vasoconstriction syndrome (RCVS), Moya-Moya-angiopathy (MMA) and PACNS, all of them leading to stenosis or occlusion of medium-sized intracranial arteries [14, 18]. Single center cohort studies of PACNS patients are limited by relatively small sample sizes. The broad clinical spectrum of PACNS and all its differential diagnoses cannot be easily mirrored adequately in small patient cohorts and comparisons between different studies are inevitably biased. However, these limitations could only be overcome by either large, multicenter prospective trials or large retrospective register studies. A selection bias in the clinical decision for or against brain biopsy might impact the clinical characteristics and histopathological findings. The good safety data on extended stereotactic brain biopsies in our cohort also might be influenced by the selection of the patients for biopsy, which means caution is required when comparing our safety data with data from other PACNS-studies. A relevant limitation of our study definitely is the quite low rate of conventional angiography of the cerebral arteries (52.2%) before brain biopsy, since some differential diagnoses of PACNS such as Divry van Bogaert syndrome or RCVS can only be diagnosed by conventional angiography [19, 20]. Particularly for the differentiation of RCVS from PACNS conventional angiography with intraarterial nimodipin application is extremely helpful [20]. However, conventional angiography probably would not have been of additional diagnostic value in most of our cases, since most patients presented with small vessel disease. Different forms of PACNS seem to exist with either at least predominant or even isolated affection of small vessels or vice versa predominant or even isolated affection of medium-sized vessels, as can be seen in the quite low rates of pathological findings in conventional angiography (0–33%) in the former scenario and comparable low rates of pathological findings in brain biopsy in the latter form (0–25%) [1, 6, 13, 14, 21].

In retrospect, one may criticize, that some of the alternative diagnoses in our patients such as PRES, APS, Ehrmann–Sneddon’s syndrome, tumefactive MS and neurotuberculosis with secondary vasculitis could have been made without brain biopsy, when following a clearly defined and stepwise diagnostic algorithm [15, 17]. However, cases sometimes present quite atypical or disease-defining biomarkers or specific laboratory tests might be ambiguous or false-negative initially or even during a longer course, so that there is a need for brain biopsy at the end. Nevertheless, we want to point out, that before obtaining brain biopsy in possible or suspected PACNS a clearly defined and stepwise diagnostic algorithm with extensive investigations always should be applied [15, 16].

We conclude that stereotactic brain biopsy in patients with suspected PACNS should not be delayed, when all the non-invasive diagnostic tests before—following a strict and extensive algorithm—do not lead to a definite diagnosis. Stereotactic biopsy should include meningeal, superficial cortical as well as intralesional specimens. Aiming at acute MRI lesions might improve diagnostic yield for positive diagnoses in general. It is a safe and minimally invasive procedure that directly impacts clinical decision making and leads to a definite diagnosis in 61% of patients.
